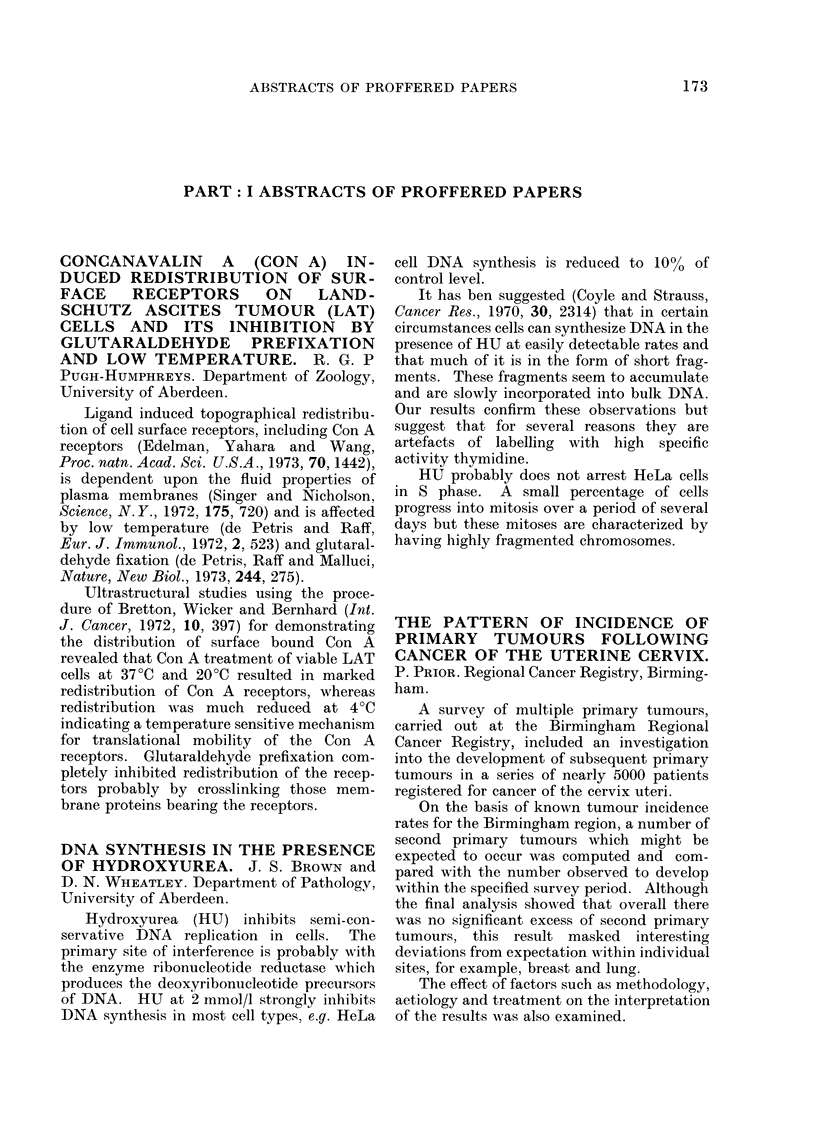# Proceedings: Concanavalin A (con A) induced redistribution of surface receptors on Land-Schutz ascites tumour (LAT) cells and its inhibition by glutaraldehyde prefixation and low temperature.

**DOI:** 10.1038/bjc.1974.132

**Published:** 1974-08

**Authors:** R. G. Pugh-Humphreys


### PART : I ABSTRACTS OF PROFFERED PAPERS

**CONCANAVALIN A (CON A) INDUCED REDISTRIBUTION OF SURFACE RECEPTORS ON LANDSCHUTZ ASCITES TUMOUR (LAT) CELLS AND ITS INHIBITION BY GLUTARALDEHYDE PREFIXATION AND LOW TEMPERATURE.** R. G. P PUGH-HUMPHREYS. Department of Zoology, University of Aberdeen.

Ligand induced topographical redistribution of cell surface receptors, including Con A receptors (Edelman, Yahara and Wang, *Proc. natn. Acad. Sci. U.S.A.*, 1973, **70**, 1442), is dependent upon the fluid properties of plasma membranes (Singer and Nicholson, *Science, N.Y.*, 1972, **175**, 720) and is affected by low temperature (de Petris and Raff, *Eur. J. Immunol.*, 1972, **2**, 523) and glutaraldehyde fixation (de Petris, Raff and Malluci, *Nature, New Biol.*, 1973, **244**, 275).

Ultrastructural studies using the procedure of Bretton, Wicker and Bernhard (*Int. J. Cancer*, 1972, **10**, 397) for demonstrating the distribution of surface bound Con A revealed that Con A treatment of viable LAT cells at 37°C and 20°C resulted in marked redistribution of Con A receptors, whereas redistribution was much reduced at 4°C indicating a temperature sensitive mechanism for translational mobility of the Con A receptors. Glutaraldehyde prefixation completely inhibited redistribution of the receptors probably by crosslinking those membrane proteins bearing the receptors.